# ProPr54 web server: predicting σ^54^ promoters and regulon with a hybrid convolutional and recurrent deep neural network

**DOI:** 10.1093/nargab/lqae188

**Published:** 2025-01-07

**Authors:** Tristan Achterberg, Anne de Jong

**Affiliations:** Department of Molecular Genetics, Groningen, Biomolecular Sciences and Biotechnology Institute, University of Groningen, Nijenborgh 7, 9747 AG Groningen, the Netherlands; Department of Molecular Genetics, Groningen, Biomolecular Sciences and Biotechnology Institute, University of Groningen, Nijenborgh 7, 9747 AG Groningen, the Netherlands

## Abstract

σ^54^ serves as an unconventional sigma factor with a distinct mechanism of transcription initiation, which depends on the involvement of a transcription activator. This unique sigma factor σ^54^ is indispensable for orchestrating the transcription of genes crucial to nitrogen regulation, flagella biosynthesis, motility, chemotaxis and various other essential cellular processes. Currently, no comprehensive tools are available to determine σ^54^ promoters and regulon in bacterial genomes. Here, we report a σ^54^ promoter prediction method ProPr54, based on a convolutional neural network trained on a set of 446 validated σ^54^ binding sites derived from 33 bacterial species. Model performance was tested and compared with respect to bacterial intergenic regions, demonstrating robust applicability. ProPr54 exhibits high performance when tested on various bacterial species, highly surpassing other available σ^54^ regulon identification methods. Furthermore, analysis on bacterial genomes, which have no experimentally validated σ^54^ binding sites, demonstrates the generalization of the model. ProPr54 is the first reliable *insilico* method for predicting σ^54^ binding sites, making it a valuable tool to support experimental studies on σ^54^. In conclusion, ProPr54 offers a reliable, broadly applicable tool for predicting σ^54^ promoters and regulon genes in bacterial genome sequences. A web server is freely accessible at http://propr54.molgenrug.nl.

## Introduction

Sigma factors are a class of proteins that are required for transcription initiation in bacteria (1). In prokaryotes, transcription initiation is carried out by the RNA polymerase (RNAP) holoenzyme with an associated sigma factor subunit recognizing the promoter DNA sequence (1). Each type of sigma factor has a unique consensus sequence, or binding motif, that it recognizes. The most common sigma factor for housekeeping genes (σ^70^) has a consensus sequence at -10 base pairs (bp) upstream of the transcription start site, and another at -35 bp (2), whilst σ^54^, a less common sigma factor has its consensus sequence at -12 and -24 bp (3). The discrepancy is due to structural differences between the two sigma factors and their associated RNA polymerase holoenzymes (4). The RNAP-σ^54^ holoenzyme forms a ‘closed promoter complex’, with a different binding surface to the ‘open promoter complex’ of the RNAP-σ^70^ holoenzyme. Additionally, the RNAP-σ^70^ holoenzyme is competent for transcription, whereas upon binding to the promoter, the RNAP-σ^54^ holoenzyme requires enhancer binding proteins to convert it to an ‘open’ complex that can actively transcribe (5).

σ^54^ is an alternative sigma factor that transcribes a subset of genes involved in nitrogen-regulation (6), flagella biosynthesis (7), motility and chemotaxis (8), among others (9–11). σ^54^-dependent promoters are studied in detail for many bacteria, such as *Escherichia coli* (12), *Salmonella typhimurium* (13) and *Pseudomonas aeruginosa* (10) (see Table 1). A study on *P. aeruginosa* has shown that targeting of σ^54^ using a molecular roadblock can be used to combat virulence, prolonging survival of an infected organism, as gene transcription of virulence-associated phenotypes, such as motility, are reduced (14). Binding to the -24-consensus sequence was targeted, thereby blocking the binding and gene transcription of the RNAP holoenzyme.

**Table 1.** (**A**) List of organisms included in the positive training set and how many motifs were included from each organism, and (**B**) list of organisms included in the negative training set and how many intergenic region sequences were included from each

(A) Number of motifs		(B) Number of non-motifs	
Organism	Motifs	Organism	Non-motifs
*Pseudomonas putida* (26)	49	*Bacillus cereus* (8)	3569
*Geobacter sulfurreducens* (27)	44	*P. putida* (26)	3331
*S. typhimurium* (13)	44	*Pseudomonas fluorescens* (40)	2916
*E. coli* (28)	42	*S. typhimurium* (13)	2668
*Vibrio cholera* (29)	32	*E. coli* (28)	2537
*Yersinia pseudotuberculosis* (30)	29	*Y. pseudotuberculosis* (30)	2474
*B. cereus* (8)	21	*Clostridioides difficile* (35)	2073
*Vibrio parahaemolyticus* (31)	20	*G. sulfurreducens* (27)	1772
*Listeria monocytogenes* (32)	20	*Enterococcus faecalis* (41)	1538
*Lactobacillus plantarum* (33)	19	*V. cholera* (29)	1443
*Agrobacterium tumefaciens* (34)	16	*Chlamydia trachomatis* (38)	406
*C. difficile* (35)	15		
*Clostridium scatologenes* (36)	15		
*Clostridium botulinum* (36)	15		
*Bacillus subtilis* (26)	13		
*Leptospira interrogans* (37)	12		
*Pantoea agglomerans* (3)	6		
*Caulobacter crescentus* (3)	6		
*Klebsiella pneumoniae* (3)	4		
*Clostridium tetani* (36)	3		
*C. trachomatis* (38)	3		
*Clostridium novyi* (36)	3		
*Campylobacter coli* (36)	2		
*Erwinia amylovora* (39)	2		
*P*.*aeruginosa* (3)	2		
*Clostridium sporogenes* (36)	2		
*Vibrio* *alginolyticus* (3)	1		
*Klebsiella* *oxytoca* (3)	1		
*Klebsiella* *aerogenes* (3)	1		
*Enterobacter* *cloacae* (3)	1		
*Proteus vulgaris* (36)	1		
*Clostridium* *perfringens* (3)	1		
*Alcaligenes* *faecalis* (3)	1		

Correct identification of σ^54^-dependent promoters in (pathogenic) bacteria could aid our understanding of regulatory mechanisms, and thereby potentially assist in fighting infections. As the discovery of binding sites using methods such as Chromatin Immunoprecipitation sequencing (ChIP-seq) is laborious and expensive, and in light of the availability of high throughput sequencing, *in silico* detection of σ^54^ binding sites has become a topic of interest within bioinformatics.

*In* *silico* prediction of σ^54^ binding sites is often carried out to assist in the discovery of the sigmulon. Current tools do not provide accurate enough predictions across bacterial genomes, which may lead to inaccurate results. Methods based on position frequency matrices, such as FIMO, are the most used classic method for finding and predicting patterns in DNA sequences, and the σ^54^ regulon (15). Other attempts at creating tools for the prediction of σ^54^ binding sites are machine learning model based, including iProm-Sigma54 (16), iPro54-PseKNC (17) and the tool of Liu *et al.* (18). However, these tools are not suitable for elucidation of the σ^54^ regulon in whole bacterial genomes, as they either provide a high number of false positives, or only function if pre-defined promoter sequences are given. An accurate, reliable and widely applicable prediction tool for σ^54^ binding sites is therefore highly needed. Researchers should be able to use such a tool for assisting in the experimental validation of σ^54^ binding sites. For example, researchers that have run a knockout experiment on σ^54^ could accurately check the differentially regulated genes intergenic regions for a σ^54^ binding site or validate ChIP-seq or ChIP-chip results.

Deep learning has previously been used for various biological sequence-dependent classification tasks, such as promoter prediction (16,19–23). For more modern machine learning-based DNA pattern mining, convolutional neural networks (CNNs) proved to be most suitable as shown by Koo *et al.* (24). As discussed by Yue *et al.*, they are particularly suited for recognition of patterns in complex spatial data, making them a good fit for DNA sequence analysis (25). Additionally, recurrent neural network layers, such as long short-term memory (LSTM) layers, can add additional performance boosts when analyzing long-ordered sequences. Current shortcomings in developing prediction models for σ^54^ binding sites are the validation strategy and datasets employed by the models. Rigorous and independent validation is a cornerstone of robust predictive modeling. Several studies have attempted to validate their models using a subset of the training data that the algorithm has not seen during training. While this approach, known as hold-out validation, shows how well a model can learn its own data, it is not representative of the model’s general ability to treat data different from the training set. This is especially important when considering the practical use and validity of a model. Other issues are the amount and diversity of training data. For example, a model trained on a dataset with an overrepresentation of one organism may not be effectively applicable to data from unrelated organisms.

In this study, we have developed a CNN with a bidirectional long short-term memory layer (BiLSTM), which helps capture sequential dependencies in the data. The model is trained on a compilation of experimentally determined σ^54^ binding sites from a range of bacteria, including human and plant pathogens. The model is benchmarked using leave-one-group-out cross-validation to test for generalization ability. We also compare our model to previously published methods, such as iProm-Sigma54 and the tool of Liu *et al.* and demonstrate that it has by far superior performance compared with a range of novel genomes. In addition, we tagged all proteins in the UniProt database that are homologous to proteins known to be a σ^54^ regulon member. Through a combination of regulon proteins and σ^54^ bindings sites, we can effectively define the σ^54^ regulon in new genomes.

## Materials and methods

### Dataset generation for prediction of σ54 binding sites

The choice of a training set is an integral and crucial part of training an accurate machine learning model. In this study, a compilation of experimentally determined σ^54^ binding sites was curated, with sequences from 36 organisms (3,8,13,26–41). Datasets were selected from papers that provided experimental, inferred, and predicted σ^54^ binding sites. Experimental evidence was usually gathered by ChIP, inferred evidence was gathered by investigations into differentially expressed genes upon knockdown of *rpoN* and inferring binding sites from overlapping ChIP-seq data from other organisms and computational predictions were also reported by some workers [papers with experimental evidence (13,29–31,34), computational evidence (33,37) and datasets that contain both (8,26–28,32,35,38,39)]. Subsequently, short sequences containing binding motifs were elongated to 62 bp based on genome context. Genome files (obtained from http://genome2d.molgenrug.nl/) were read, a reverse complement was made and a match for the motif was searched for within the forward and reverse complement. If a match was found, the sequence was elongated on both sides to a length of 62 bp. Sequences longer than 62 bp were shortened, whilst keeping the motif region as central as possible by using the σ^54^ motif. As negatives, or non-motif-containing sequences, intergenic regions were used. This contained promoter, as well as non-promoter, sequences, from 11 organisms. Intergenic regions were obtained from http://genome2d.molgenrug.nl/ and are based on translation start sites. Transcription start site data for many organisms are not yet available, as are accurate prediction tools. Therefore, intergenic regions based on translation start site data are the most effective for removing coding regions from the dataset. To ensure that no σ^54^ binding motifs were present in the negatives, σ^54^ binding sites present in the positive set were removed, and orthologs of the *E. coli* σ^54^ regulon, obtained using BLASTP (42), were removed. Additionally, as *P. fluorescens*, *E. faecalis* and *C. trachomatis* do not have a high number of validated σ^54^ binding sites, we removed intergenic regions in the negative set containing a match to the *E. coli* σ^54^ binding motif. Matches were determined using the *E. coli* σ^54^ probability matrix from PRODORIC (MX000100), using a threshold of nine to determine σ^54^ binding sites (43).

### Feature generation

The final training sequence length of both motif-containing and non-motif-containing sequences was 50. To show the binding motif in different contexts, such as at the start, middle and end of the sequence, a sliding window approach was used. A window of three bases was used on the motif-containing sequences for data augmentation (44). A sequence length of 50 was selected to show context around the binding site. For the negative dataset, intergenic regions shorter than 50 were omitted, and a sliding window of three bases was used to capture the whole length of the regions in different contexts. The sequences were then one-hot encoded, with each nucleotide represented in its own four-element binary vector.

### Model selection

To determine which model architecture would perform best on the data, we ran hyperparameter optimizations on a range of machine learning models using the Optuna framework (45). Model architectures included a random forest, a support vector machine (SVM), a simple neural network, a CNN and a CNN including a BiLSTM.

For the random forest model, we optimized the maximum depth of the trees (max_depth), so that the model could capture sufficient complexity while avoiding overfitting. Additionally, we tuned the minimum number of samples required to split a node (min_samples_split) and the minimum number of samples per leaf (min_samples_leaf). These both control the growth of the tree, preventing overfitting by limiting the number of samples that create new branches.

For the SVM, the regularization parameter (C) was optimized between 0.00 001 and 10 000, to achieve the best trade-off between margin maximization and error minimization. The kernel type was chosen from rbf and linear to explore both linear and non-linear decision boundaries. The gamma parameter for the rbf kernel was also optimized between 0.00 001 and 10 000 to control the influence of each training example on the decision boundary.

In the simple neural network, we varied the number of hidden layers between one and four. A higher number of layers allows for increased capacity to learn complex patterns, but also risks overfitting. The number of units per layer was selected between 32 and 256 to ensure sufficient representational power. Similarly, a larger number of units per layer allows for increased learning capacity, but higher risk of overfitting. Dropout layers were also introduced and dropout rates optimized between 0.1 and 0.5 to prevent overfitting, ensuring that the model generalizes well to unseen data. The final output layer used a sigmoid activation function.

For the CNN, we varied the number of convolutional layers (n_conv_layers) between one and three, experimenting with different filter sizes and kernel widths to optimize feature extraction from the input data. Similarly, we introduced dropout layers and varied dropout rates for both convolutional and dense layers to regularize the model. The CNN–BiLSTM hybrid model built upon the CNN architecture, introducing a BiLSTM layer to capture sequential dependencies within the data. We set a range of 32 to 256 units for the LSTM layer.

For each neural network, the models were compiled with a binary cross-entropy loss function. In this case, the binary classification is either 0 (negative sequence), not a motif containing sequence, or 1 (positive sequence), a motif containing sequence. The *adam optimizer* was used to optimize the binary cross-entropy (46). Model performance was evaluated based on its binary accuracy. For the dense and convolutional layers, the rectified linear unit activation function was used due to its ability to mitigate the vanishing gradients problem (47).

To further optimize the training process for the neural networks, we implemented a learning rate scheduler and early stopping mechanism. A learning rate scheduler was employed to adjust the learning rate dynamically during training. The learning rate was optimized between 0.1 and 0.5. This ensures that the model continues to make improvements during later stages of training, when the validation loss plateaus. The patience for the learning rate reduction, which defines the number of epochs with no improvement before the learning rate is decreased, was tuned between 3 and 10 epochs. This helped prevent premature convergence or stagnation in training.

Additionally, early stopping was implemented to halt training when the model’s validation loss stopped improving, with the patience parameter optimized between 5 and 20 epochs. This prevented overfitting by stopping training before the model began to degrade in performance. A batch size of 32 was used to train the models, and the neural networks were set to run for 100 epochs.

All Optuna studies were set to run for 100 trials. The best model hyperparameters were selected based on the models mean Matthews correlation coefficient (MCC) score over the seven bacteria present in the leave-one-group-out cross-validation set. Due to high data imbalance in the dataset (after sliding window implementation 1221 660 non-sigma 54 binding sequences to 2225 sigma 54 binding sequences), we implemented a random under sampling strategy for the majority class. For the random forest and SVM models, we used a ratio of 1:8, meaning eight non-sigma 54 binding site sequences for each sigma 54 binding site sequence. For the neural network models, we used a ratio of 1:40, as these ratios were empirically found to achieve the best results. For more detailed information on the architecture and hyperparameters used in this study, see the GitHub https://github.com/annejong/ProPr54.

### Model validation on unseen data

Intergenic region files from seven bacteria (*B. cereus*, *E. coli*, *G. sulfurreducens*, *P. putida*, *S. typhimurium*, *V. cholera* and *Y. pseudotuberculosis*) were retrieved from http://genome2d.molgenrug.nl/. These bacteria were selected, as they had the highest number of experimentally validated binding sites, and a diverse GC content. *E. coli*, *P. putida*, *S. typhimurium*, *V. cholera* and *Y. pseudotuberculosis* share the class of Gammaproteobacteria, whilst *B. cereus* and *G. sulfurreducens* are in the phyla of Bacillota and Thermodesulfobacteriota, respectively. The respective GC contents of the intergenic regions from each bacterium used in the validation set were as follows: *B. cereus*: 29.93%, *E. coli*: 41.14%, *G. sulfurreducens*: 53.31%, *P. putida*: 54.75%, *S. typhimurium*: 41.82%, *V. cholera*: 41.61% and *Y. pseudotuberculosis*: 40.08%.

A new model was trained for predictions on each bacterium, with data from the bacterium omitted from the training features, a method known as leave-one-group-out cross-validation. We also ran an error analysis on the model predictions, analyzing motif positions, GC content and motif scores. Significance scores were calculated using a Mann–Whitney *U*-test.

Additionally, proteins encoded by genes containing a σ^54^ motif in their upstream region were BLASTed against the UniProt database to make a UniProt regulon database. The database was curated from genomes highlighted in Table 1, with genomes without locus-tags, or genome files, being omitted. Predictions on features of genomes with no compilation of σ^54^ binding sites were carried out. The UniProt regulon database was then BLASTed against the genomes of interest to predict putative regulon members based on protein similarity the members of the regulon in the new genome. The predictions were then examined for how many of the machine learning predicted regulon members are also predicted to be a regulon member by protein similarity.

MEME was used to input positive predictions from ProPr54 for *B. cereus* to generate a sequence motif. MEME searched for three motifs with a minimum width of six and a maximum width of 30. TOMTOM (48) was used to search in the PRODORIC database for motif matches (43).

### Performance comparison with other models

For optimization of the model, the following parameters were used:

- Precision = TP/(TP + FP),
- Recall = TP/(TP + FN),
- Accuracy = (TP + TN)/(TP + TN + FP + FN) and
- MCC = (TP*TN-FP*FN)/SQR[(TP + FP) *(TP + RN) *(TN + FP) *(TN + FN)].

In these formulas, TP, FN, TN and FP are true positives, false negatives, true negatives and false positives, respectively. The performance of the model was compared with the prediction model of Liu *et al.*, iProm-Sigma54 and FIMO. Recall, precision, MCC and *F*1 score were compared on the intergenic regions of seven bacteria.

As each model requires different input, for Liu *et al.* the data was prepared as instructed on https://github.com/maqin2001/PromotePredictor, with feature selection performed on the training data in WEKA. The model was then retrained. Features from the seven bacteria were created with a sliding window with a k-mer length of 81 and step size of three over the intergenic regions. No feature selection was performed on the intergenic region features, as the *InputMappedClassifier* tool in WEKA selected matching features for testing. Model performance was evaluated by comparing the model output with the experimentally validated σ^54^ binding sites, similarly to our model.

For iProm-Sigma54, the features were created with a sliding window with k-mer length of 81 and step size of three. The iProm-Sigma54 model weights and source code were retrieved from github.com/Shujaatmalik/iProm-Sigma54.

Testing iPro54-PseKNC performance on a whole genome is not feasible as the web server does not output all results, but only a small portion of the input sequences. FIMO performance was also tested on the bacteria’s intergenic regions, with a cut-off value of 1^−5^ taken, as this produced a comparable number of results to other tools.

### -24/-12 σ^54^ binding site promoter dataset

To generate a promoter set containing σ^54^ binding sites at the -24/-12 position in relation to the transcription start site, we utilized the σ^54^ binding motifs identified from our training dataset. Sequences were elongated upstream and downstream of the binding site to the required length (81 for Liu *et al.* and iProm-Sigma54, and 50 for ProPr54) in order to position the binding site at the -24/-12 position. For FIMO, sequences of 81 nucleotides in length were used and a cutoff of 1^−5^ was taken for positive predictions.

### σ^54^ regulon protein database

Using our carefully curated σ54 regulon proteins (Supplementary Table S1) as a foundation, we systematically identified and tagged all proteins within the UniProt database that exhibited similarity, thereby constructing an expanded database encompassing σ^54^ regulon proteins. Unidirectional BLASTP with a stringent e-value cutoff of 10-e09 was used to identify putative regulon proteins in the UniProt database.

### Web server and GitHub

For ease of use, we developed a web server, available at http://sigma54.molgenrug.nl/, which hosts the developed CNN model and can give extra information following the prediction. Required are a DNA file in FASTA format and optimally a GFF annotation file. The web server runs the prediction and will show genes predicted to contain a σ^54^ motif in their upstream region. It will highlight the motif, and provide its position within the k-mer, and the overall sequence. The web server also indicates which strand the motif appears on. Optionally, in case a dataset is larger and contains non-intergenic regions, a GFF file can be included. The web server will automatically determine the intergenic regions based on the GFF, and only predict on those regions. A prediction is also made based on protein homology, using our curated σ^54^ regulon proteins database. The overlap between ProPr54 predictions, and protein homology predictions, is determined and displayed. The WebLogo of the discovered motifs will be displayed on the web server. Results will also provide a functional classification of the genes, clusters of orthologous groups and KEGG pathways based on our FACoP genome annotation program (49). FACoP also returns operon predictions and unified gene names, which is used to include all genes of an operon in the predicted regulon. A prediction of the σ^54^ regulon can be run on a whole genome in a matter of minutes. Example files that can be used on the web server, along with scripts for generating your own features and training the model with our model architecture, can be found at https://github.com/annejong/ProPr54.

## Results & discussion

### Model architecture optimization

**Figure 1.**
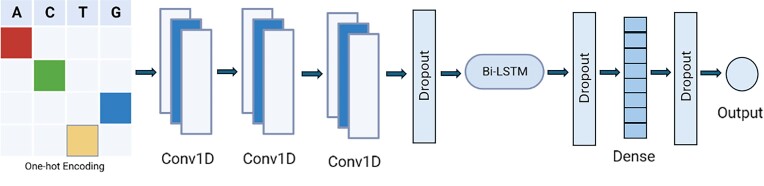
Graphical illustration of the ProPr54 model. The DNA sequence is first one-hot encoded and input to the convolutional layers (Conv1D) which act as motif scanners across the input matrix. Outputs from the convolutional layers are fed into the BiLSTM with a dropout in between. The BiLSTM captures the spatial dependencies and context within the sequence by processing the sequence in both forward and backward directions. After the BiLSTM layer, features are then fed through a dense layer with dropouts in between to mitigate overfitting. The output is transformed into a probability distribution over the two classes by the sigmoid activation function.

To determine which model architecture is best suited for accurate prediction of σ^54^ binding sites, we compared the performance of five model architectures on intergenic regions of seven bacteria. The model architectures included a random forest, a SVM, a simple neural network, a CNN and a CNN with a BiLSTM layer (Figure 1). All models were evaluated using a leave-one-group-out cross-validation scheme and underwent hyperparameter optimization using the Optuna framework (45).

**Figure 2.**
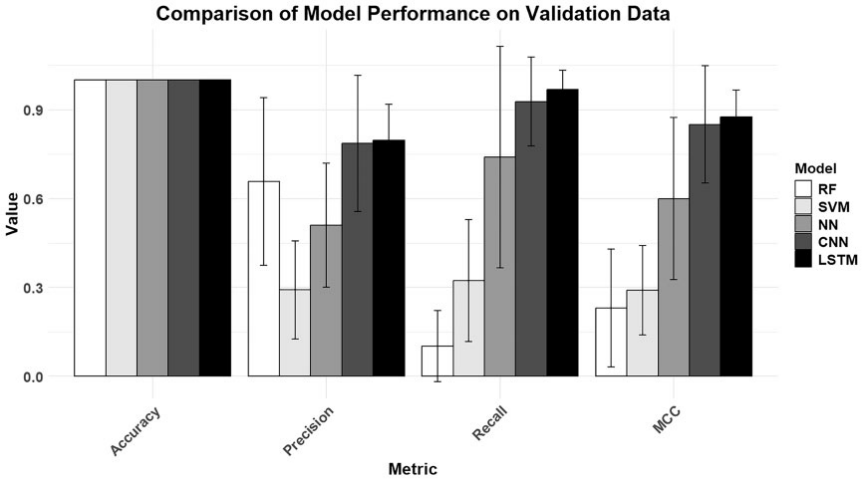
Model architecture performance on the validation set. The BiLSTM-CNN (LSTM) obtains the best precision, recall and MCC scores compared with other available model architectures.

Due to high class imbalance, accuracy across all models was 1. The BiLSTM neural network (from now on referred to as ProPr54) was able to achieve the highest performance across all metrics, with a precision of 0.8, a recall of 0.97 and an MCC of 0.88 (Figure 2). The random forest and SVM were unable to achieve comparable mean MCC scores (0.23 and 0.29), and the neural network achieved a mean MCC of 0.6. The absence of a BiLSTM layer in the CNN decreased the mean MCC to 0.85, also leading to a higher variance in performance between different bacterial species. A higher overall recall was achieved by the models compared with precision.

These scores show a clear preference for CNNs in the prediction of σ54 binding sites, with the inclusion of a BiLSTM layer further enhancing model performance. Non-neural network models like random forests and SVMs were not able to achieve robust prediction capabilities. These models lack the ability to process DNA sequences sequentially and do not inherently capture positional information or interactions between distant elements within the sequence. Neural networks on the other hand can process input data in a way that preserves the order of features, which may be important for DNA binding motifs. The addition of the CNN allows for the detection of local motifs, such as the σ^54^ binding sites. Additionally, CNNs can recognize motifs no matter where they appear in the sequence, whereas simple neural networks are required to learn each possible position of a motif separately. The BiLSTM layer adds the ability for the model to capture dependencies between distant nucleotides and is designed for handling sequences where the order of data is important. This could allow the model to understand the surroundings of the σ^54^ binding site better, such as the downstream transcription start site.

We additionally ran an error analysis, examining the GC content, motif positions and position weight matrix (PWM) motif scores of the incorrect and correct predictions of ProPr54 (Supplementary Figure S1). For both motif scores and GC content, there was a highly significant difference (*P* < 0.001) between false positives and correct negatives. Similarly, for the motif scores, there were highly significant differences both between correct positives and false negatives and correct negatives and false positives.

These findings indicate that the model may have difficulties predicting outliers, for example in unexpected GC content or motif score ranges. For the motif scores, it seems that sequences with higher PWM motif scores tend to be predicted positive, and those with lower scores tend to be predicted negative. The errors could also be caused by a skewed dataset. As there is only a finite number of available experimentally determined binding sites, and this number varies per organism, a bias in the models’ performance toward organisms with more motifs may be present. The high average recall of the model, coupled with lower average precision, may indicate that ProPr54 is capturing binding sites that were not able to be captured by experimental techniques. This could be in part due to the general inactivity of some promoters under the control of σ^54^, or because the experimental conditions were not able to induce differential activity of σ^54^ promoters. Use of *insilico* tools such as ProPr54 can therefore capture σ^54^ binding sites active only in certain conditions.

### Model comparisons on independent dataset

**Figure 3.**
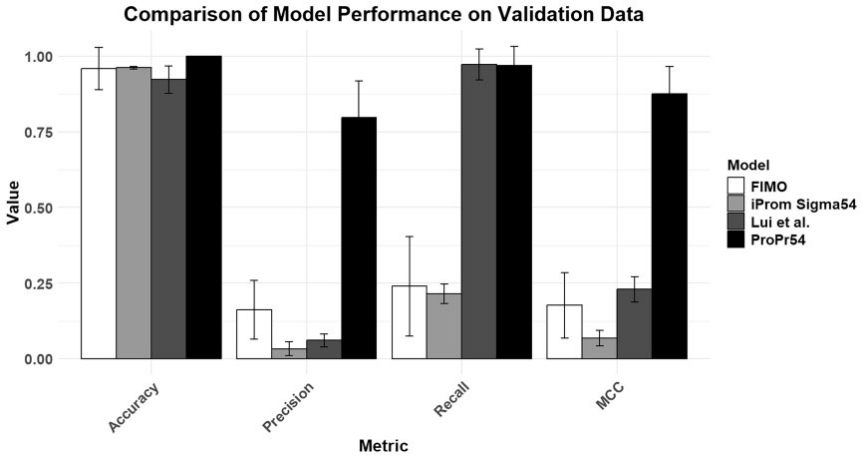
Model Performance on the validation set. ProPr54 obtains the best MCC and *F*1 scores compared with other available methods. The second highest performing method in *F*1 score is FIMO (15), with Liu *et al.* (18) achieving the second highest MCC. iProm-Sigma54 (16) scored the lowest in MCC and *F*1. The Recall of ‘Liu *et al.*’ is very high due to the extreme number of false positives.

To compare available models and motif scanning software, we benchmarked our model with that of Liu *et al.*, iProm-Sigma54 and FIMO on the intergenic regions of seven bacteria included in the original training set, obtained from Genome2d. The model of Liu *et al.* and iProm-Sigma54 are both machine learning models trained on σ^54^ binding sites, and made for identification of σ^54^ promoters, whilst FIMO is a widely used motif scanning tool. Our model achieved a mean precision of 0.8, a recall of 0.97 and an MCC of 0.88 (Figure 3). The next best available prediction tool was the tool of Liu *et al.* (MCC = 0.23), which uses a random forest. Liu *et al.* achieved a recall comparable to ProPr54 of 0.97, but it provided a high rate of false positives, with a precision of 0.06. FIMO achieved the second highest MCC (0.18), using a PWM to scan the DNA sequence. iProm-Sigma54 achieved the lowest MCC of 0.07. Accuracy is high across the board due to the class imbalance. These results show that our tool highly outperforms other available prediction tools.

The use of a random forest by Liu *et al.* instead of a CNN could be the reason for their lower performance. Random forests lack the ability to treat the DNA sequences sequentially, as they handle input features independently. Additionally, they are less capable of capturing interactions between elements that are not close to each other within the sequence, which convolutional and BiLSTM layers are designed for. It is also likely that the threshold is set too low for the detection of positives, as their model predicted many positives, which included the true positives, but led to a high number of false positives. The dataset of Liu *et al.* additionally only contained 222 sequences, compared with our overall dataset of 45 900 sequences, before data augmentation. This allowed our model to see a much wider diversity of sequences, possibly allowing it to generalize better to unseen data. For validation of their model, Liu *et al.* also tested model performance on unseen genomes. The true positives of the validation data were however derived from another prediction tool, PromScan, which utilizes a PWM approach similar to FIMO. Based on the performance of FIMO, it can be assumed that using PWM-based predictions as true positives would likely not yield accurate validation, potentially leading to overestimation of model performance. As our model utilizes a robust, comprehensive validation set, we were therefore able to develop our model to perform well on unseen genomes.

The iProm-Sigma54 model used a CNN, however their dataset comprised overwhelmingly of *E. coli* promoter sequences. Overall, their dataset comprised of 2998 sequences, with their negative set including solely *E. coli* sequences. This means that use of entire intergenic regions, for which we do not know the promoter regions, does not yield comparable MCC scores to our model. The use of mainly *E. coli* sequences also makes the generalization capability of the model onto other species poor. Additionally, the evaluation of their model was not done on any new genomes. A 5-fold cross-validation was used, which does show model performance on data similar to its training data, it does not show high generalization ability.

Both iProm-Sigma54 and Liu *et al.* did also not use a sliding window to generate training data, showing the σ^54^ binding site in different contexts. This is especially important when predicting unseen data. Input sequences for which the location of σ^54^ binding sites, or transcription start sites is not known (as is the case in many bacteria), could contain the binding site at any location. It is therefore important for the model to be able to recognize σ^54^ binding sites in different positions in the sequence. For ProPr54, we ran predictions using identical model architecture trained on data that did not undergo sliding window data augmentation, which showed far worse model performance (Supplementary Figure S2).

In FIMO the use of a PWM generally constrains the motif to the DNA binding site. It does therefore not take into account the surrounding sequence, and simply applies a matrix to each window. Whilst this is an effective and simple method, it can miss sequences that are too dissimilar to the sequences the matrix was based on. It can also overestimate the number of binding site containing sequences, as sequences that look similar to the binding site, but are not in the correct biological context can be falsely predicted as positives.

Our model displayed much superior performance mainly due to a large and biologically diverse dataset and a robust validation strategy and model selection. We included 36 organisms in the training data, and model performance was evaluated on a diverse set of bacteria, importantly all including previously verified σ^54^ binding sites. Additionally, our leave-one-group-out cross-validation strategy coupled with hyperparameter optimization tests the model in a biologically and experimentally relevant scenario. Optimizing model performance across the seven organisms that the model had not been previously trained allows for the selection of the model with the highest accuracy coupled with the best generalization capabilities. This is especially important considering the diversity and size of bacterial genomes.

### Prediction of σ^54^ promoters with perfect positioning

**Figure 4.**
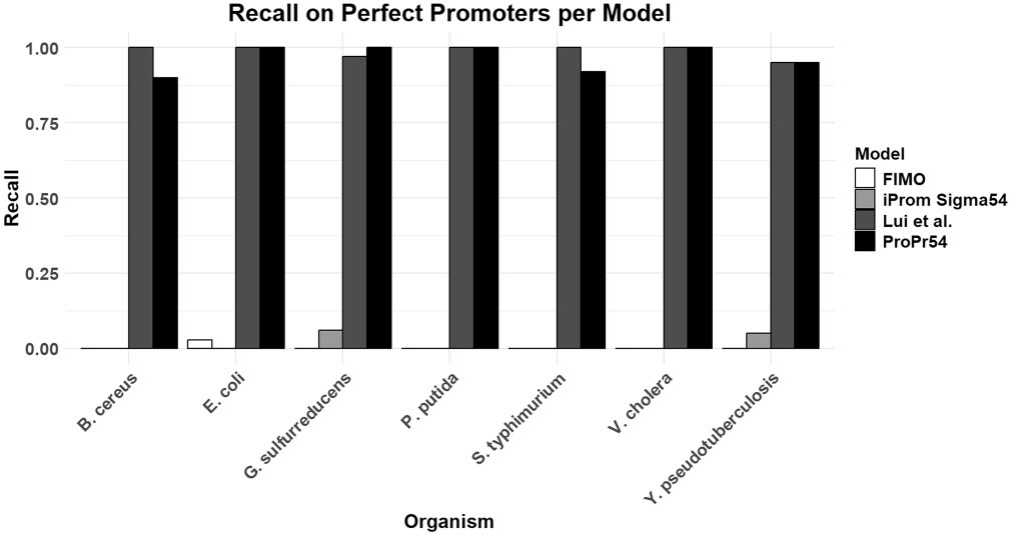
Recall on promoters with the σ^54^ binding site at the -24/-12 position in relation to the transcription start site. FIMO and iProm-Sigma54 are both unable to recall the σ^54^ binding sites, whilst both Liu *et al.* and ProPr54 have high levels of recall across all organisms.

To show that the performance discrepancy between the models is not solely due to the sliding window employed on the validation data, we tested our model on promoters with the σ^54^ binding site at the -24/-12 position. This places the σ^54^ binding site at the correct position regarding the transcription start site and shows the model’s ability to predict σ^54^ binding sites under perfect conditions. Results show that both ProPr54 and the model of Liu *et al.* can consistently recall the true positives with the σ^54^ binding site at the -24/-12 position (Figure 4). The model of Liu *et al.* achieved a mean recall of 0.99. ProPr54 achieved an average recall of 0.97. iProm-Sigma54 and FIMO are both unable to recall the experimentally verified binding sites, with both models achieving a recall of 0 on five out of the seven organisms.

**Figure 5.**
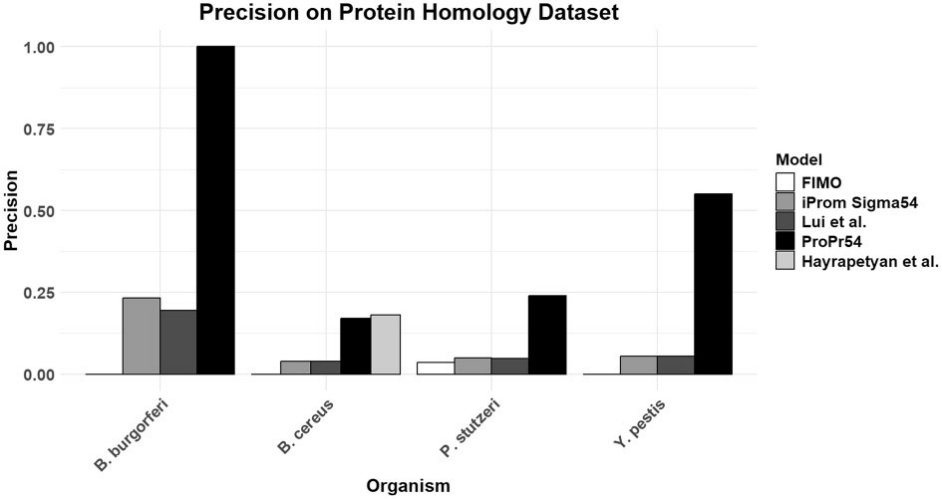
Overlap percentage between regulon inferred from protein similarity, and regulon inferred from ProPr54 promoter predictions on four different organisms. *B*.*cereus* (H) is the calculated overlap percentage based on the regulon experimentally discovered by Hayrapetyan *et al.* (8).

This indicates that iProm-Sigma54 and FIMO would not be suitable for use in cases where the transcription start sites are known, whereas both the model of Liu *et al.* and ProPr54 are able to identify the promoters. As datasets with reliable transcription start sites with the σ^54^ binding sites at the -24/-12 position are not available, we did not include any negatives in this prediction. The model of Liu *et al.* could in this case also have a poor precision due to a high number of false positives. iProm-Sigma54 showing low scores indicates that their model may have been trained on data with the σ^54^ motif in a different position. As their model was trained mostly on *E. coli* promoters, and does not perform well in our test, it is likely that the *E. coli* promoters did not correspond to transcription start sites. The low performance of FIMO is again likely due to the use of a PWM, which may not be able to effectively capture relevant information around the binding motif.

### Prediction of σ^54^ promoters in organisms with no experimentally verified σ^54^ sigmulon

In order to show that our tool is generally applicable, we predicted σ^54^ regulons in four bacteria *Yersinia pestis*, *Borrelia burgdorferi*, *Pseudomonas stutzeri* and *B. cereus*. Three of the genomes (*Y. pestis*, *B. burgdorferi* and *P. stutzeri*) did not have a published compilation of σ^54^ binding sites. Our σ^54^ promoter prediction tool was used to screen the intergenic regions of these bacteria. To enable a fair prediction, we used a model in which *B. cereus* was excluded from the training dataset. Secondly, putative σ^54^ regulon proteins were predicted based on unidirectional BLASTP against our σ^54^ regulon database. The precision on the predictions was used as the performance metric, with a correct prediction defined as a positive prediction within a gene that was part of the putative σ^54^ BLAST regulon. We also included the experimentally verified σ^54^ binding sites from Hayrapetyan *et al.*

Across all organisms ProPr54 achieved the highest precision, with precisions of 1, 0.17, 0.24 and 0.55. FIMO and the model of Liu *et al.* achieved similar precisions across models, with average precisions of 0.09 and 0.08. The experimental binding sites of Hayrapetyan *et al.* scored a precision of 0.18 on *B. cereus*, with ProPr54 achieving a precision of 0.17 and iProm-Sigma54 and Liu *et al.* a precision of 0.04, respectively (Figure 5).

Comparisons on *B. cereus* show that only ProPr54 was able to achieve a precision comparable to that of an experimentally validated dataset. This indicates that ProPr54 is the closest to being able to replicate, by prediction, promoters that contain the σ^54^ binding site and are homologous to genes that are under control of σ^54^. The extremely high performance on *B. burgdorferi* for ProPr54 is because the model only predicted one promoter to contain a σ^54^ binding site. The *B. burgdorferi* genome is smaller than the other organisms, having only 422 intergenic regions compared with 4320 in *B. cereus*, leading to a lower number of predictions. Precision levels across other organisms are similar between iProm-Sigma54 and Liu *et al.*, indicating that there may be a level of random guessing, whereas ProPr54 displays higher precision levels across all organisms. FIMO was unable to obtain high precision levels across all organisms, which is unexpected considering it achieved the second highest average precision level in Figure 3. This may indicate that performance among these organisms may be lower compared with those used in the validation set. It is unlikely that the low precision is due to the BLAST dataset, as the validation data from Hayrapetyan *et al.* achieved the highest precision in *B. cereus*.

### Identified motifs

**Figure 6.**
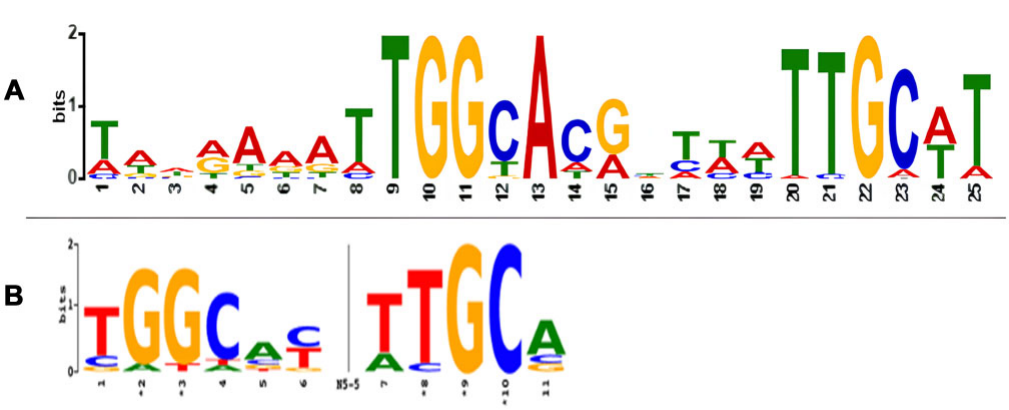
(**A**) WebLogo generated by MEME on upstream regions of the predicted σ54 regulon of *B. cereus*, with a threshold of 0.5. (**B**) Known σ54 motif of *E. coli*; PRODORIC MX000100.

**Figure 7.**
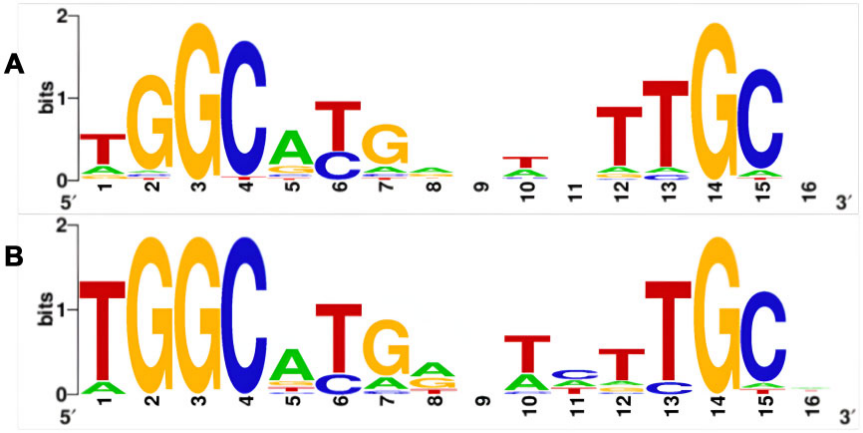
WebLogo generated by MEME on upstream regions of *Y*.*pestis* intergenic regions (**A**) based on predicted σ54 promoters, and (**B**) based on putative σ54 protein regulon members.

On the basis of upstream regions of the predicted σ^54^ regulon, a WebLogo was created to visualize the motif detected in *B. cereus* by our tool and compared this with the known *E. coli* σ^54^ binding motif from PRODORIC (Figure 6 and 7) (43,50). This matches MX000100 with a *P*-value of 2.31e^−09^, meaning that it has a high likelihood of matching the *E. coli* σ^54^ binding motif. This shows that our prediction model was able to effectively recognize the σ^54^ binding motif and even make it more specific for *B. cereus*. It can also reproduce it in an independent dataset, even when the organism had not been included in training (results not shown).

### Web server example: *K. pneumoniae*

With this example, we demonstrate the performance of the ProPr54 web server on the prediction of the σ^54^ promoters and regulon of *K. pneumoniae*. From NCBI we downloaded the DNA sequence (.fna) and the annotation file (.gff) of the reference genome *K. pneumoniae* subsp*. pneumoniae* HS11286 (including the six pKPHS plasmids) and *K. pneumoniae* VAKPC309 which is only available as Whole Genome Sequence (WGS) which consists of 217 contigs (see supplementary materials *Klebsiella_pneumoniae.zip*). From both strains the WGS and the associated annotation file was submitted to the ProPr54 web server. The results showed that the predicted σ^54^ binding site TGGCA(T/C)(G/A)nnnnTTGCA resembled the expected pattern in both *K. pneumoniae* strains. From the results the intersection table of genes with predicted σ^54^ promoter in the upstream region and showed similarity with UniProt sigma54 regulon proteins was combined with the predicted Operons. Using the assumption that all genes in the predicted operon are under the control of the same promoter we identified 88 genes for the reference genome strain HS11286 and 95 genes for WGS genome strain VAKPC309, which contain a σ^54^ promoter and showed similarity with the reference σ^54^ regulon proteins database (Table 2). Because also generalized gene names are given by the web server, we observed that in one case the *hypABCDE* operon was predicted as two operons (*hypA* and *hypBCDE*, operon_2138 and operon_2140, respectively), but most likely this is one operon with only a σ^54^ promoter upstream of the *hypA* gene (Supplementary Table S2).

**Table 2.** Web server results of *K. pneumoniae* subsp. *pneumoniae* HS11286 reference genome and a random strain *K. pneumoniae* VAKPC309 (for details see Supplementary Table S2)

*K. pneumoniae* strain	σ^54^ promoters	σ^54^ regulon proteins	Intersection including operons
HS11286	43	398	88
VAKPC309	51	389	95

## Conclusions

We present and introduce ProPr54, the first tool able to accurately identify the σ^54^ regulon, leveraging its capability to recognize σ^54^ promoters. Our approach incorporates a CNN with a BiLSTM layer and three convolutional layers. In sharp contrast to previous tools, ProPr54 showcases an accurate performance on independent data, attributed to its unique model architecture. By integrating a BiLSTM, ProPr54 surpasses existing tools that either omit CNNs or lack recurrent modeling. This ProPr54 model utilizes a larger and more diverse dataset than previous training sets. As a result, compared with previous models, the performance of the model has advanced in the following ways: (i) The increased variability of the data enables ProPr54 to generalize more robustly to novel genomes. Results underscore ProPr54 as the current state-of-the-art model for predicting σ^54^ promoters in entire genomes, providing users with consistently accurate outcomes. (ii) Moreover, ProPr54 incorporates predictions based on protein similarity and operons, presenting users with an overlap analysis between σ^54^ promoter predictions and those derived from protein similarity and operon structures. This holistic approach enhances the reliability of the regulon predictions. (iii) The versatility of ProPr54 extends to its applicability across a wide range of organisms, making it a valuable tool for researchers studying σ^54^ binding sites or regulon members. The importance of σ^54^ was already shown by Lloyd *et al.* (14), to combat virulence in *P. aeruginosa*. In our study, we showed that ProPr54 can predict the σ^54^ regulon in *K. pneumoniae* that will enhance research on virulence factors in bacterial species. But ProPr54 is not limited to pathogenic bacteria because in many species’ nitrogen biosynthesis, flagella biosynthesis, motility, chemotaxis and various other essential cellular processes are under control of σ^54^.

Instead of using inaccurate PWM methods, researchers can use ProPr54 to assist in their understanding of a bacteria’s σ^54^ regulon, or in their validation or cross-referencing of experimental σ^54^ regulon results. This high-performance model is now accessible through a user-friendly web server, ProPr54, and facilitates the submission of a list of sequences or full genome sequences allowing the model to predict the organism’s σ^54^ regulon.

## Supplementary Material

lqae188_Supplemental_Files

## Data Availability

The data underlying this article are available on Github at https://github.com/annejong/ProPr54, and on Zenodo at https://doi.org/10.5281/zenodo.14531825.
