# Supplementary figures and images for: ProPr54 web server: predicting σ54 promoters and regulon with a hybrid convolutional and recurrent deep neural network
Source: NAR Genom Bioinform. 2025 Jan 7;7(1):lqae188. doi: 10.1093/nargab/lqae188 (PMC11704786; doi:10.1093/nargab/lqae188)

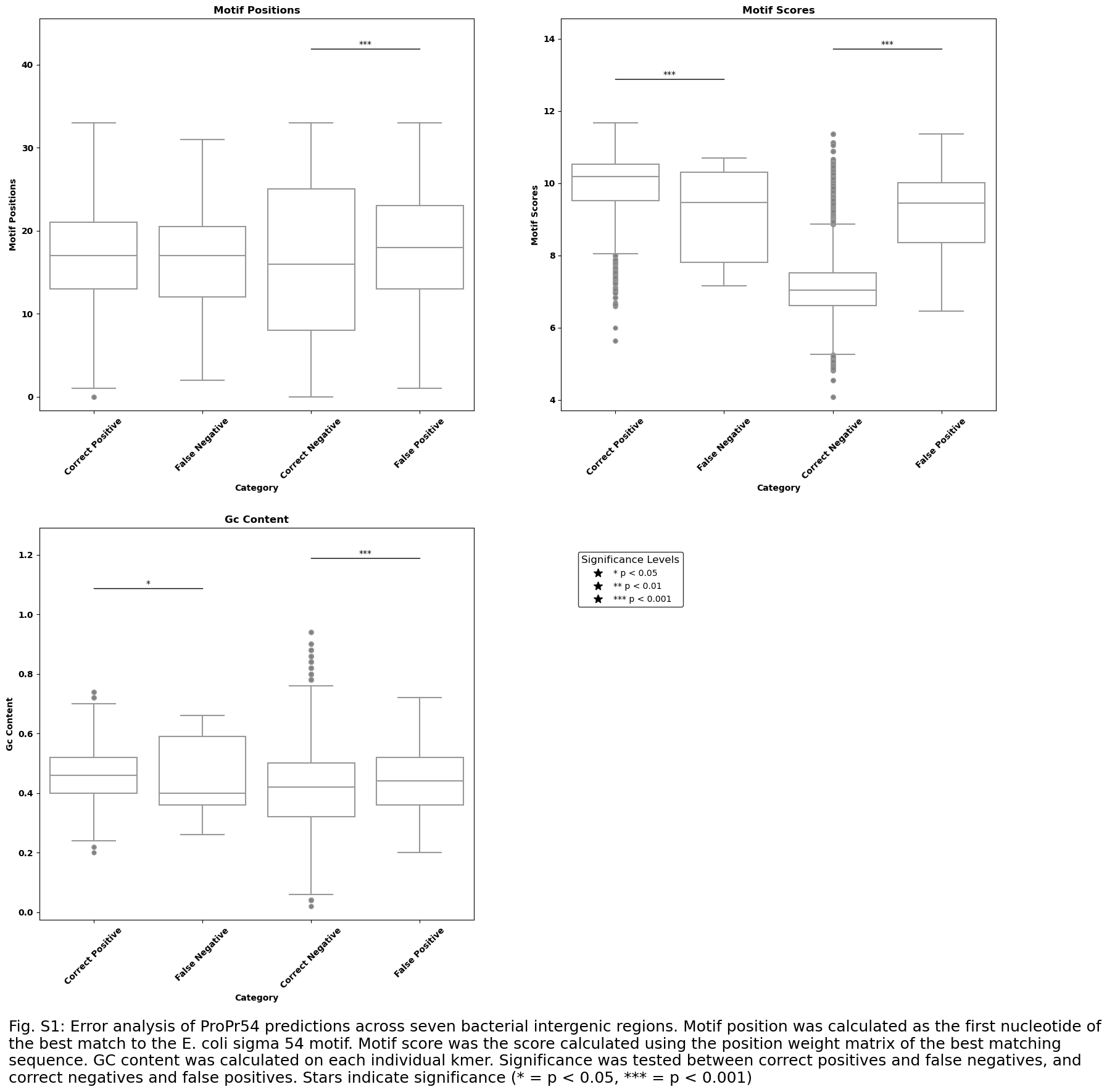

Supplement: lqae188_Supplemental_Files [file lqae188_supplemental_files.zip › Fig_S1.png]

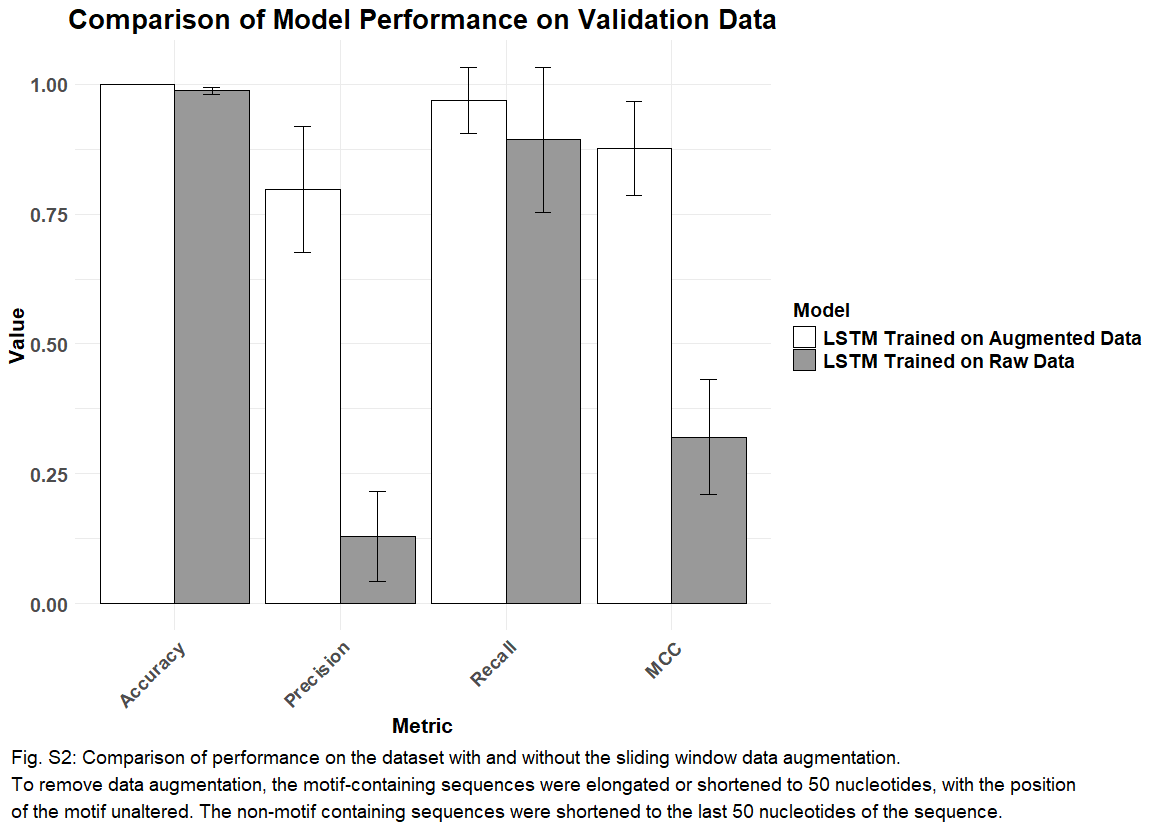

Supplement: lqae188_Supplemental_Files [file lqae188_supplemental_files.zip › Fig_S2.png]
